# Enhanced NMDA receptor pathway and glutamate transmission in the hippocampal dentate gyrus mediate the spatial learning and memory impairment of obese rats

**DOI:** 10.1007/s00424-024-02924-1

**Published:** 2024-02-28

**Authors:** Dingding Lv, Bin Xiao, Huaying Liu, Linping Wang, Yingshun Li, Yin Hua Zhang, Qinghua Jin

**Affiliations:** 1 Department of Physiology and Pathophysiology, College of Medicine, Yanbian University, Yanji, 133002 China; 2 Department of Physiology and Biomedical Sciences, Ischemia/Hypoxic Disease Institute, Seoul National University, College of Medicine, Seoul, Korea

**Keywords:** Obesity, NMDA receptor, Glutamate transmission, Dentate gyrus, Learning and memory, Rat

## Abstract

Obesity has been linked with the impairment of spatial memory and synaptic plasticity but the molecular mechanisms remained unidentified. Since glutamatergic transmission and NMDA receptor neural pathways in hippocampal dentate gyrus (DG) are essential in the learning and memory, we aimed to investigate glutamate (Glu) and NMDA receptor signaling of DG in spatial learning and memory in diet-induced obesity (DIO) rats. Spatial learning and memory were assessed via Morris water maze (MWM) test on control (Ctr) and DIO rats. Extracellular concentration of Glu in the DG was determined using in vivo microdialysis and HPLC. The protein expressions of NMDA receptor subunit 2B (NR2B), brain-derived neurotrophic factor (BDNF), the activation of calcium/calmodulin-dependent kinase II (CaMKII) and cAMP-response-element-binding protein (CREB) in the DG were observed by western blot. Spatial learning and memory were impaired in DIO rats compared to those of Ctr. NR2B expression was increased, while BDNF expression and CaMKII and CREB activation were decreased in DG of DIO rats. Extracellular concentration of Glu was increased in Ctr on the 3rd and 4th days of the MWM test, but significant further increment was observed in DIO rats. Microinjection of an NMDA antagonist (MK-801) into the DG reversed spatial learning and memory impairment. Such effects were accompanied by greater BDNF expression and CaMKII/CREB activation in the DG of DIO rats. In conclusion, the enhancement of Glu-NMDA receptor transmission in the hippocampal DG contributes to the impairment of spatial learning and memory in DIO rats, maybe via the modulation of CaMKII-CREB-BDNF signaling pathway.

## Introduction

Obesity is one of the most serious public health challenges of the twenty-first century due to its close associations with severe comorbidities, including cardiovascular and metabolic disorders [11]. More importantly, obesity is increasing at an alarming rate in children and adolescents, and overweight during developmental period of the youth are linked with cognitive impairments, episodic and spatial memory deficits [21, 22, 29, 16, 4, 32, 1]. This can be problematic particularly because this period is in parallel with the maturation of the hippocampus, exacerbating the central-behavior coordination. Experimentally, high-fat diet (HFD) exposure to rodents from weaning to adolescence and adulthood has been shown to induce substantial impairment of both hippocampal plasticity and hippocampal-dependent memories [22, 9, 7]. Intriguingly, different from HFD exposure from weaning to the adulthood (covering adolescence), when substantial impairment of both hippocampal plasticity and hippocampal-dependent memories occurred, HFD exposure in the adulthood did not affect memory [4, 17]. These results indicate that DIO-induced learning and memory alterations in the hippocampus is dynamic, pointing towards the prospect of potential therapeutic drug development. Thorough understandings of spatial learning and memory in the hippocampus of obese models are important in preventing abnormal cognition and memory function.

Dentate gyrus (DG) is one of the two critical regions in the hippocampus (DG and CA1-3) those are involved in spatial learning and memory in mammals by encoding and processing spatial information to the hippocampus [25, 14]. Moreover, convincing evidences indicate the impairment of spatial memory in rodent models of DIO, manifested by morphological damage, inflammation, and a decrease in neurogenesis in the DG region of the hippocampus [4, 5, 10]. Therefore, these reports strongly support the notion that the dysregulation in DG are crucial in spatial learning and memory deficits in DIO. However, the neurochemical mechanisms in the DG those are responsible for the learning and memory deficits of DIO are largely unknown.

It is well established that N-methyl-D-aspartate-type glutamatergic (NMDA) receptor -mediated signaling is important in learning and memory processes through hippocampal long-term potentiation (LTP). Activation of NMDA receptors and the consequent increase in Ca^2+^ influx in postsynaptic cells triggers the phosphorylation of cAMP-response- element-binding protein (CREB) via Ca^2+^/calmodulin- dependent kinase II (CaMKII) [3, 19]. Subsequently, CREB phosphorylation activates the transcription of brain-derived neurotrophic factors (BDNF) by binding to a cAMP response element within the gene, increase BDNF protein expression [34, 23]. Previously, results from our group indicated that Glu in the hippocampal DG is involved in spatial learning and memory impairments in Alzheimer’s rat model [27]. However, the roles of the Glu and NMDA receptor of DG in spatial learning and memory impairments in DIO have not been reported. Therefore, this study aims to investigate whether diet-induced obese rats show the phenotype of spatial learning and memory impairment and whether Glu and NMDA receptor-mediated signaling are altered in the DG of DIO rats. Combining behavioral test analysis with molecular biological experiments, we analyzed molecular mechanisms those are involved in spatial learning and memory deficits in DG of DIO rats.

## Materials and methods

### Establishment of DIO model in rats

All the experimental protocols were conducted according to the NIH Guide for the Care and Use of Laboratory Animals and the ethical regulations of Yanbian University. Humane manner was applied to minimize animal suffering and minimum numbers of rats were killed. Sprague–Dawley rats (male, 4 weeks old) were provided by Changsheng Laboratory Animal Technology (SCXK[LIAO]2000–0001, Shenyang, China).

Rats were randomly divided in two groups (similar average body weight, housed two per cage): Ctr diet group (3.82 kcal/g; 10 kcal% fat, 70 kcal% carbohydrates, 20 kcal% protein; Qianmin, Liaoning province, China) and HFD group (4.73 kcal/g; 45 kcal% fat, 35 kcal% carbohydrates, 20 kcal% protein, D12451, Research Diets Co. Ltd., New Brunswick, NJ, USA). Rats were exposed to the Ctr diet or HFD for 12 weeks (from weaning to adulthood), and body weight was monitored once per week.

### Behavioral tests

The spatial learning and memory abilities of the rats were assessed using the morris water maze (MWM) task, which included a place navigation trial and spatial probe trial. A circular pool (diameter: 170 cm; height: 60 cm) made of plastic, with the inner surface painted black, (Taimeng, China) was filled with opaque water (22 ± 2 ℃). The pool was divided equally into four quadrants, and a small circular platform (diameter: 10 cm) was placed in the center of the third quadrant and submerged 5 cm beneath the water surface. The escape latency, swimming speed, proportion of total time spent in each quadrant, and number of platform crossings were measured with tracking system software (SMART v3.0, Panlab, Spain). In the place navigation trial, hidden platform training was consecutively performed for 4 days, and each daily training session included four training trials. For each trial, rats were randomly placed into the water of one quadrant facing the wall, and were allowed to find the platform within 120 s and rest on there for 15 s. If a rat was unable to find the hidden platform within 120 s, it was led to the platform where it stayed for 15 s, and the escape latency was recorded as 120 s. The daily order of entry into individual quadrants was randomized such that all four quadrants were used once every day, and in each case the rat was given the next trial after the rest period. The average latencies of the four training trials were compared among the groups. The spatial probe trial was performed on the 5th day of the MWM test. The circular platform was removed from the pool, and the animals were placed in the water at a given location to swim freely for 120 s; then the proportion of total time spent in each quadrant and the number of platform crossings were recorded.

### Measurement of Glu concentration

Glu concentration was measured by HPLC and electrochemical detection system (HTEC-500, Eicom, Japan). Before employing the HTEC-500, a 4 mM o-phthalaldehyde (OPA) solution was made by adding 1.35 mg of OPA and 1 µl of 2-mercaptoethanol to 2.5 ml of 0.1 M K_2_CO_3_ buffer (pH 9.5) with 10% ethanol. Then, 12 μl of the standard solution (including 2 µM corresponding Glu) or the dialysate was mixed with 3 μl of 4 mM OPA solution and allowed to react for 2.5 min in a 25 °C incubator. After completing the reaction, 10 μl of the reaction mixture was applied to the SC-5ODS column (2.1 mm ID × 150 mm) in the HTEC-500. Detection was accomplished with + 600 mV Ag/AgCl electrodes. The elution buffer for Glu consisted of 0.1 M phosphate buffer, 30% methanol, and 0.5 mM EDTA (pH 6.5). The waves of Glu in the chromatograms were identified by the retention time in the standard solution, and the concentration of Glu was calculated according to the area of the amino acid wave in the standard solution.

### Microdialysis in DG

Rats were anesthetized with 10% chloral hydrate (300 mg/kg, i.p.) and placed on a stereotaxic frame (David Kopf, USA). According to the atlas of Paxinos and Watson, a guide cannula was stereotaxically implanted 1.0 mm above the DG region (coordinates: 3.2 mm posterior to the bregma, 1.6 mm lateral to the midline, and 2.5 mm ventral to the dural surface), and fixed to the skull by dental cement. The animals were individually housed with access to food and water and allowed to recover from surgery for 2 days. After recovery, the rats were anesthetized using ethyl ether and a microdialysis probe or microinjection tube was inserted through the guide cannula into the DG region and stabilized with wax. To reach the DG region, the microdialysis probe or microinjection tube was guided 1.5 mm or 1.0 mm beyond the guide shaft, and the tip of the probe was covered with a 1.5-mm length of acetate cellulose membrane (OD, 0.2 mm; cutoff, 5.0 × 10^4^ mol wt.; DM-22, Eicom, Japan).

### Western blot analysis

The hippocampal DG samples were homogenized in ice-cold lysis buffer containing 50 mM Tris (pH 7.4), 150 mM NaCl, 1% Triton X-100, 1% sodium deoxycholate, 0.1% sodium dodecyl sulfate (SDS), 1 mM PMSF, and complete protease inhibitor cocktails. The tissue homogenate solutions were centrifuged (13,000 g, 30 min, 4 °C), and the supernatants were collected and used to quantify the total protein using a Bradford Protein Assay kit. Equal amounts of protein (30 μg) for each sample were separated by SDS–polyacrylamide gel electrophoresis and transferred electrophoretically onto polyvinylidene fluoride (PVDF) membranes. The PVDF membranes were blocked with 5% (w/v) skim milk in phosphate-buffered saline (PBS) with 0.15% Tween 20 (PBST) at room temperature. After 2 h, the membranes were incubated with anti-CREB, anti-^S133^CREB, anti-^T286^CaMKII, anti-β-actin 1:1000; Cell Signaling Technology, Inc., MA, USA), anti-NR2B, anti-BDNF (1:1000; Abcam, Cambridge, UK), and anti-CaMKII (1:250; Abcam) antibodies in PBST at 4 °C overnight. The membranes were washed three times in PBST for 15 min, then incubated for 1 h with secondary antibodies (1:2000) at room temperature. After extensive washing with PBST, protein bands were visualized with the ECL Plus western blotting detection system (ComWin Biotech, Beijing, China) and quantified using the Biospectrum 615 imaging system.

### Reagent

MK-801 (St. Louis, MO, USA), an antagonist of NMDA receptor, was dissolved in modified Ringer’s solution (147 mM NaCl, 4 mM KCl, 2.3 mM CaCl_2_; pH 6.5) (60 μmol/L), then stored at 4℃. A microinjection tube was connected to the microinjection pump (ESP-64, Eicom, Japan) on the day of behavioral test, and 1 μL of the drug solution was injected into the DG 30 min before the behavioral test.

### Experimental designs and sample sizes

Experimental designs and the animals used for the experiments are: 1) Two Glu measurement groups, the Ctr (Ctr, n = 6) and DIO groups (n = 6), in which the spatial learning and memory; metabolic parameters in the serum; and Glu concentration, expression of NMDA receptor subunit 2B (NR2B) and BDNF, and activation of CaMKII and CREB in the DG region were examined; 2) Three MK-801 groups, Ctr + vehicle (Ctr + veh, n = 6), DIO + vehicle (DIO + veh, n = 6), and DIO + MK-801 (n = 6) groups, in which spatial learning and memory, and BDNF expression and CaMKII and CREB activation in the DG region, were examined.

On the day after the insertion of the microdialysis probe, the collection of dialysates from the DG region and the behavioral test were carried out under freely-moving conditions. The microdialysis probe was perfused with modified Ringer’s solution at 1.5 μl/min using a microinfusion pump (ESP-64, Eicom, Japan), and the dialysate from the DG region was automatically collected by a fraction collector (EFC-82, Eicom, Japan) every 10 min at 4 °C. Three consecutive dialysate samples were collected to measure the concentration of Glu.

After the end of the spatial probe trial of the MWM test, the animals were anesthetized with isoflurane and sacrificed by rapid decapitation. Blood was collected immediately and the brain was removed. The blood samples were centrifuged (200 0 g for 10 min at 4℃) to obtain serum, and the levels of total cholesterol (CHO), triglycerides (TG) and glucose were determined with an automated biochemical analyzer (AU480, BeckmanCoulter, USA). In addition, the concentrations of leptin and insulin in the serum were measured using ELISA kits (SEKR-0033 and SEKR-0051, Solarbio, China) according to the manufacturer’s instructions. The hippocampal DG region was quickly dissected out from the brain on an ice-cold surface, and western blot analysis was carried out.

### Statistical analysis

Data are expressed as the mean ± SEM, and statistical analysis was performed with GraphPad Prism 7.0 software. Independent-samples *t*-test was used to compare the metabolic parameters of the serum, the basal level of Glu, percentage of time spent in target quadrant, number of platform crossings, and expressions of proteins in the “Glu measurement groups,” and the chance the animals’ performance was random was assessed (for spatial memory) through a one-sample *t*-test. Other data were analyzed using ANOVA with repeated measures and post hoc analysis. A value of *P* < 0.05 was considered statistically significant.

## Results

### Effects of HFD exposure on body weight and metabolism

**Fig. 1:**
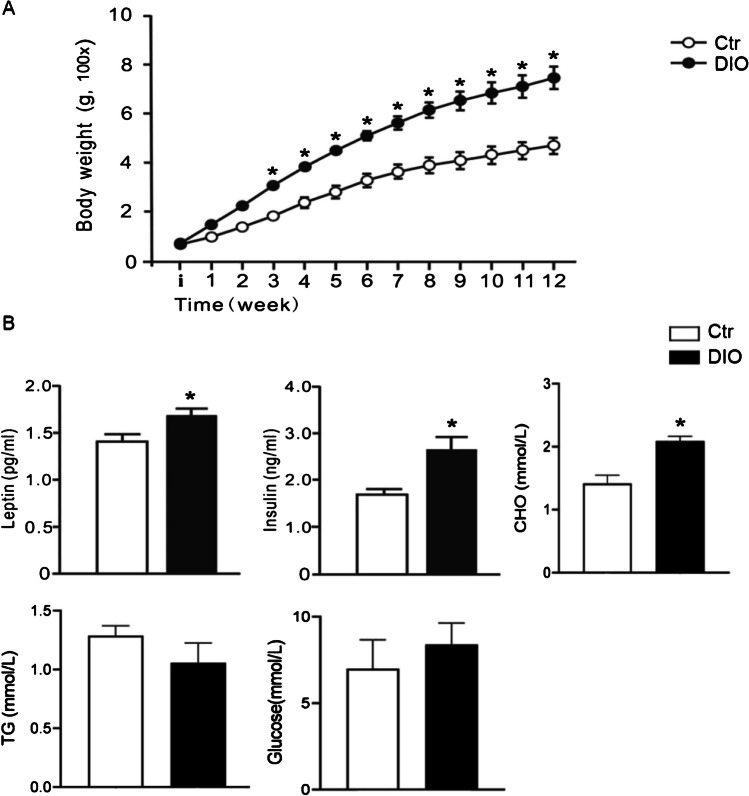
Effects of HFD exposure on body weight and metabolism. **A** Body weight; **B** metabolic parameters in the serum. Data are mean ± SEM; CHO, TG and Glucose n = 5/ Ctr group; n = 9/ DIO group. Leptin and insulin n = 4/group. ^*^ *P* < 0.05 compared with Ctr group

Body weight between the Ctr and DIO groups were not different before the start of diet exposure (70.59 ± 6.93 g in Ctr group and 72.63 ± 2.96 g in DIO group) (t_(12)_ = 0.27, *P* > 0.05). 12 weeks of diet exposure induced significant changes for diet (F_(1,12)_ = 27.93, *P* < 0.05), time (F_(12,144)_ = 252.4, *P* < 0.05), and an interaction between these factors (F_(12,144)_ = 14.18, *P* < 0.05) (Fig. 1A and Table 1), indicating that the rate of weight gain were differed between the groups. Post hoc analyses indicated that the HFD rats were significantly heavier than Ctr diet rats from week 3 of diet exposure (*P* < 0.05), and on the 12th week, DIO rats were 58% heavier than the Ctr diet rats.

**Table 1:** Effect sizes of body weight, leptin, insulin and CHO

Name	Variable	Cohen’s d	f-value
Body weight	Time (week)	–	254.20
	DIO	–	27.93
Leptin	DIO	2.20	–
Insulin	DIO	2.18	–
CHO	DIO	2.57	–

The metabolic parameters in the serum were measured at the time of sacrifice (Fig. 1B). Compared with the Ctr group, leptin (t_(6)_ = 3.110, *P* < 0.05), insulin (t_(6)_ = 3.080, *P* < 0.05), and CHO (t_(12)_ = 4.812, *P* < 0.05) in the serum of DIO rats were significantly increased (Table 1). However, the levels of TG and glucose in the serum were not affected by the HFD (TG: t_(12)_ = 0.967, *P* > 0.05; glucose: t_(12)_ = 0.676, *P* > 0.05).

### Spatial learning and memory were impaired in DIO rats

**Fig. 2:**
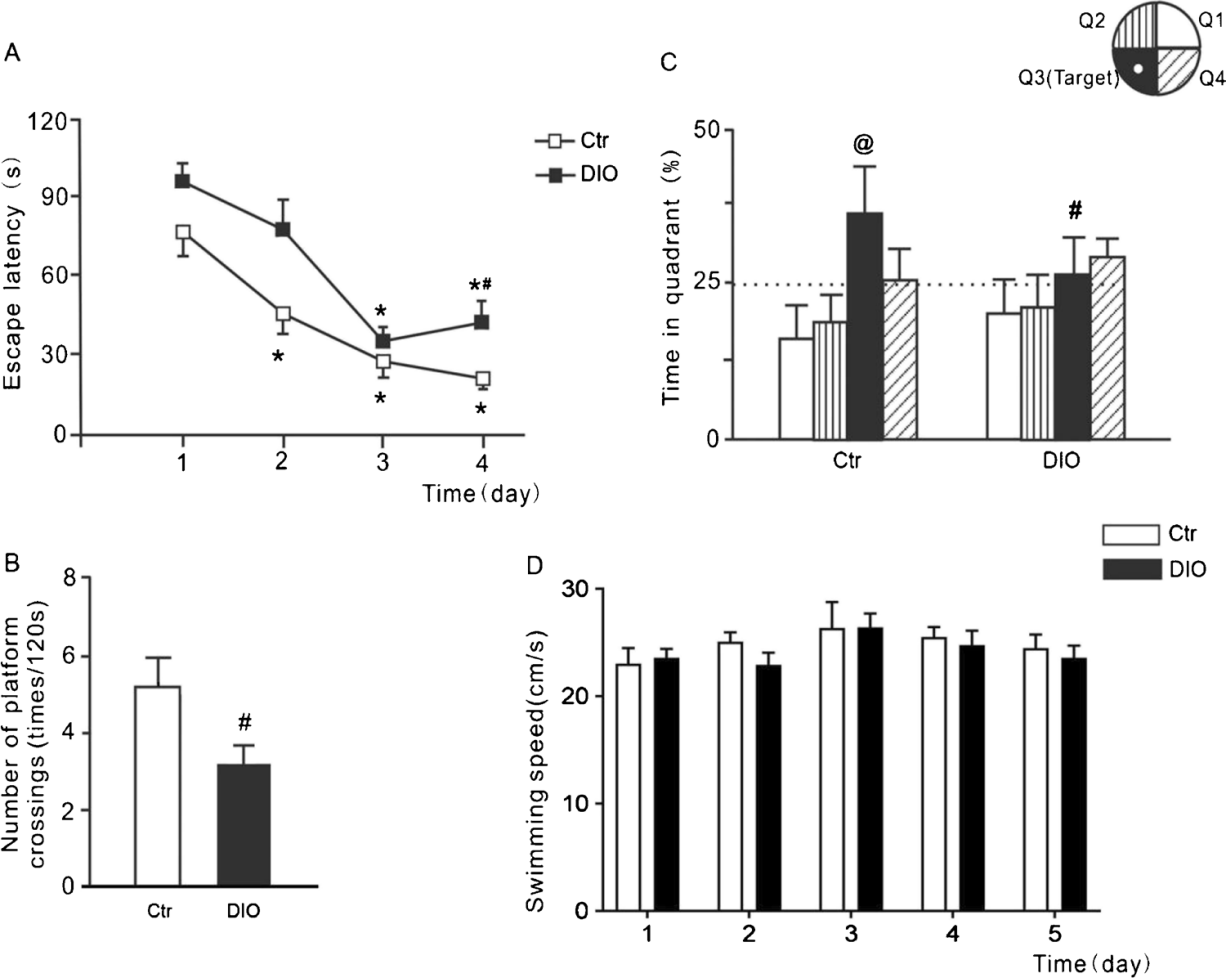
Changes in spatial learning and memory abilities in DIO rats. **A** Escape latency in the place navigation trial of the MWM test; **B** proportion of total time spent in each quadrant and **C** number of platform crossings in the spatial probe trial of the MWM test; **D** swimming speed on each day of the MWM test. Data are mean ± SEM; n = 7/group. * P < 0.05 compared with first day, ^#^ *P* < 0.05 compared with Ctr group, ^@^* P* < 0.05 compared with 25% chance level for each quadrant

During the 4 days of the place-navigation trial in the MWM test, the escape latency decreased in both the Ctr and DIO groups (F_(3,24)_ = 12.36, *P* < 0.05 in the Ctr group and F_(3,24)_ = 10.51, *P* < 0.05 in the DIO group); however, on the 4th days, escape latency was significantly longer in the DIO group than in the Ctr group (t_(12)_ = 2.181, *P* < 0.05) (Fig. 2A and Table 2), indicating that there was spatial learning impairment in the DIO group.

**Table 2:** Effect sizes of escape latency, time in quadrant and number of platform crossings

Name	Variable		Cohen’s d	f-value
Escape latency	Time (week)		–	42.37
	DIO		–	4.85
Time in quadrant	DIO		1.60	–
Number of platform crossings	DIO		1.38	–

In the spatial probe trial of the MWM test, rats in the Ctr group showed significant bias towards the target quadrant (t_(6)_ = 4.644, *P* < 0.05), while rats in the DIO group failed to show a preference for the target quadrant (t_(6)_ = 0.944, *P* > 0.05) (Fig. 2B). Compared with the Ctr group, the percentage of time spent in the target quadrant (t_(12)_ = 3.003, *P* < 0.05, Fig. 2B) and the number of platform crossings (t_(12)_ = 2.58, *P* < 0.05, Fig. 2C) were markedly reduced in the DIO group (Table 2), indicating spatial memory deficiency occurred in the DIO. Figure 2D illustrates the swimming speed of both the Ctr and DIO groups across the training days, and data analysis revealed no significant difference between the groups (F(_4,60_) = 0.5953, *P* > 0.05).

### Glu levels were enhanced and NR2B expression was increased in the DG of DIO rats

**Fig. 3:**
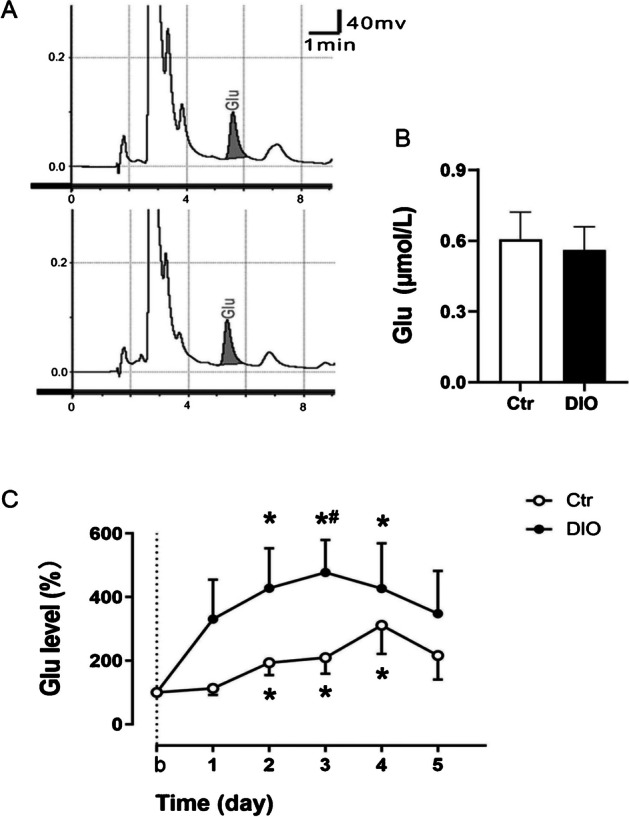
Changes in Glu concentration in the hippocampal DG during MWM test in DIO rats. **A** Typical HPLC-ECD chromatograms of the Glu in the dialysate of the DG before the start of behavioral tests in Ctr group (above) and DIO group (below); **B** Basal levels of Glu concentration in the DG; **C** Glu levels in the hippocampal DG during the MWM test expressed as a percentage of basal level. Data are mean ± SEM; n = 7/group. ^*^P < 0.05 compared with basal level, ^#^*P* < 0.05 compared with Ctr group

The extracellular concentration of Glu in the hippocampal DG was determined by in vivo brain microdialysis and HPLC techniques. In the present study, the “basal level” was taken as the Glu concentration obtained before the start of behavioral tests, and the Glu level in the DG during the MWM test was expressed as a percentage of the basal level. As shown in Fig. 3A and B, there was no difference in basal levels of Glu in the DG between the Ctr and DIO groups (t_(12)_ = 1.179, *P* > 0.05) (Table 3).

**Table 3:** Effect sizes of Glu level

Name	Cohen’s d
Level of Glu	0.63

**Fig. 4:**
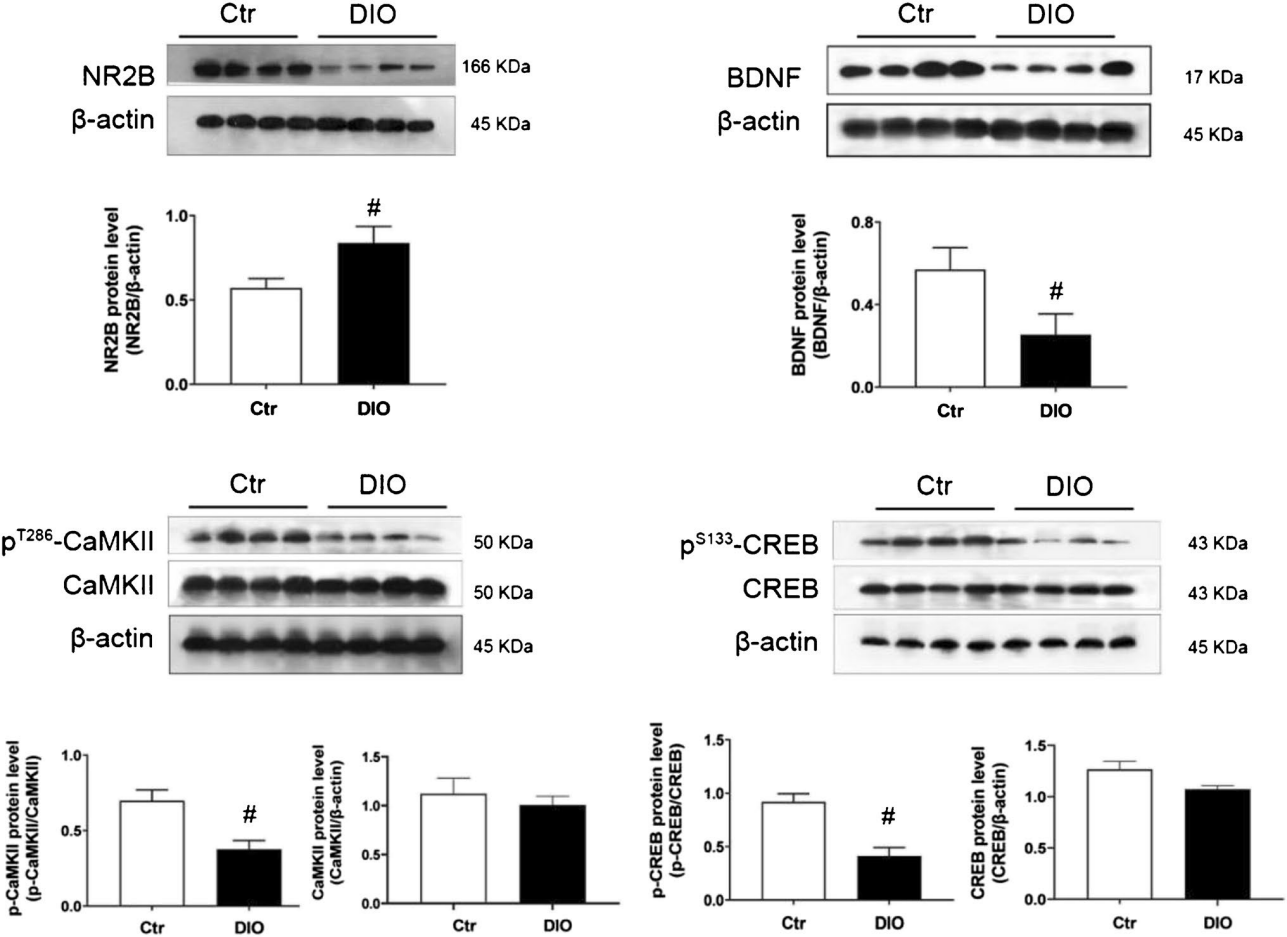
Changes in expression of NR2B (**A**) and BDNF (**B**) and activation of CaMKII (**C**) and CREB (D) in the DG of DIO rats. Data are mean ± SEM; n = 4/group. ^#^*P* < 0.05 compared with Ctr group

Glu levels in the DG were significantly increased on the 2nd to 4th days of the MWM training in both the Ctr and DIO groups (F_(5,36)_ = 2.055, *P* < 0.05 in Ctr group and F_(5,36)_ = 2.368, *P* < 0.05 in DIO group); however, on the 3rd days, Glu levels in the DG were significantly higher in the DIO group than the Ctr group (3rd day: t_(72)_ = 3.093, *P* < 0.05) (Fig. 3C), suggesting that the Glu response in the DG during the spatial learning process was markedly disturbed in DIO rats. In addition, the expression of NR2B (t_(6)_ = 4.747, *P* < 0.05) was significantly increased in the DG of the DIO group rats compared with that of the Ctr group rats (Fig. 4A and Table 4).

**Table 4:** Effect sizes of NR2B, BDNF, p-CaMKII and p-CREB

Name	Cohen’s d
NR2B	3.36
BDNF	3.07
p-CaMKII	2.47
p-CREB	3.31

### BDNF expression and CaMKII and CREB activation in the DG were inhibited in DIO rats

The protein levels of CaMKII, CREB, BDNF, CaMKII-phosphorylation (^T286^CaMKII), and CREB-phosphorylation (^S133^CREB) in the hippocampal DG were measured by western blot analysis. The activities of CaMKII and CREB were measured via the ratios of phosphorylated to total proteins, respectively. Compared with the Ctr group, the activation level of CaMKII (t_(6)_ = 3.486, *P* < 0.05), CREB (t_(6)_ = 4.683, *P* < 0.05) and expression of BDNF (t_(6)_ = 4.338, *P* < 0.05) of DIO groups were significantly decreased (Fig. 4B, C, D and Table 4).

### Effects of MK-801 on spatial learning and memory in DIO rats

**Fig. 5:**
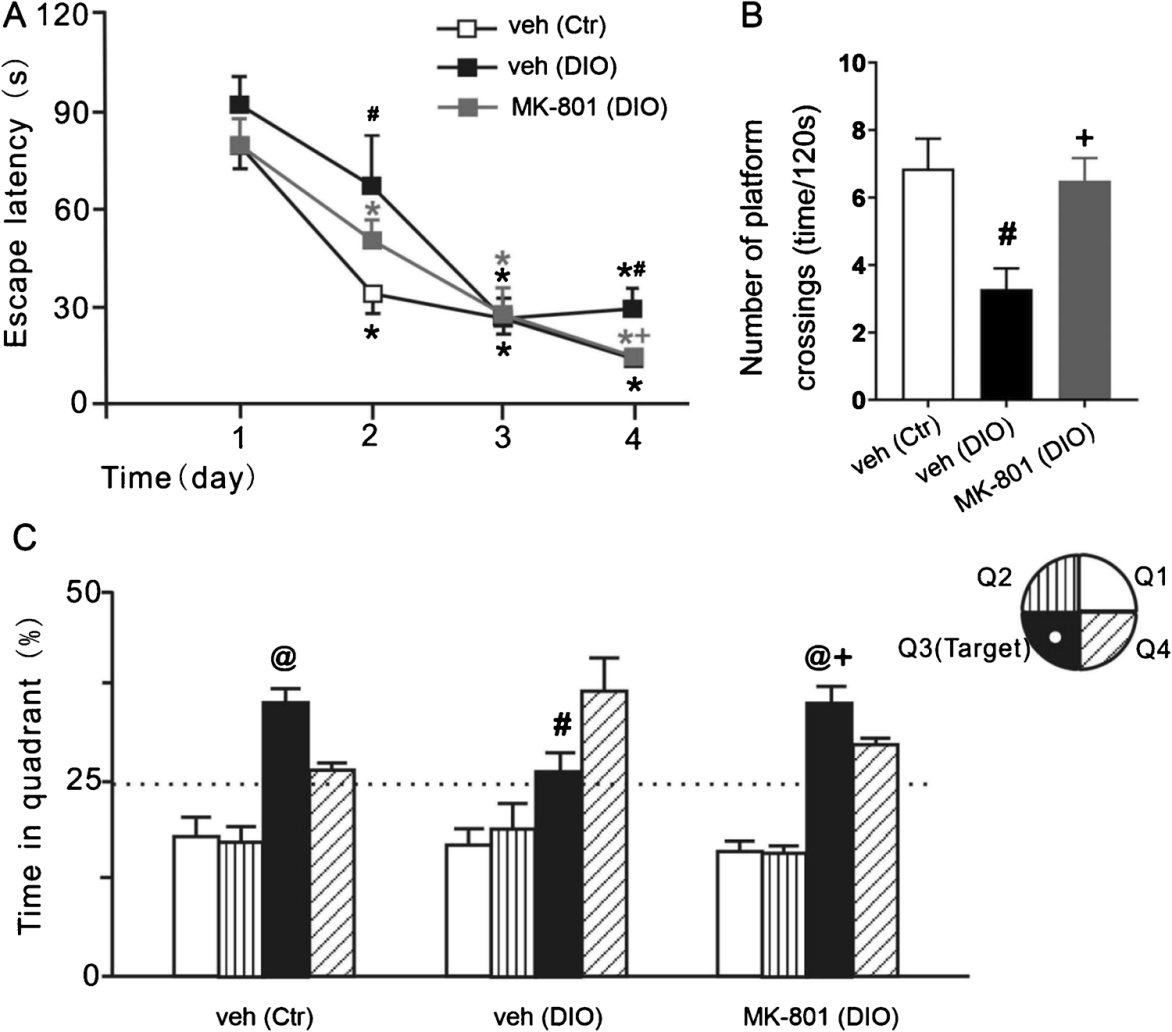
Effect of MK-801 on spatial learning and memory in DIO rats. **A** Escape latency in the place navigation trial of the MWM test; **B** number of platform crossings and **C** proportion of total time spent in each quadrant in the spatial probe trial of the MWM test. Data are mean ± SEM; n = 6/group. ^*^ *P* < 0.05 compared with first day, ^#^ *P* < 0.05 compared with control + vehicle group, ^+^*P* < 0.05 compared with DIO + vehicle group, ^@^*P* < 0.05 compared with 25% chance level for each quadrant

To further investigate whether the increase in Glu in the DG was involved in DIO-induced spatial learning and memory impairments via the activation of NMDA receptors, MK-801 (an antagonist of the NMDA receptor) was microinjected into the DG, and spatial learning and memory in DIO rats were observed. During the 4 days of the place-navigation trial of the MWM test, each treatment group’s escape latency gradually decreased during repeated training (F_(2,9)_ = 21.69 in Ctr + veh group, *P* < 0.05; F_(2,11)_ = 12.46 in DIO + veh group, *P* < 0.05; F_(2,9)_ = 22.81 in DIO + MK-801 group, *P* < 0.05); however, the escape latency of the DIO + MK-801 group on the 4th day was significantly reduced compared with that of the DIO + veh group (t_(15)_ = 3.395, *P* < 0.05) (Fig. 5A and Table 5).

**Table 5:** Effect sizes of escape latency, time in quadrant and number of platform crossings

Name	Variable	Cohen’s d	f-value
Escape latency	Time (week)	–	49.47
	DIO	–	2.86
Number of platform crossings	MK-801	2.05	–
Time in quadrant	MK-801	1.63	–

In the spatial probe trial of the MWM test, rats in the Ctr + veh group and DIO + MK-801 group showed a significant bias towards the target quadrant (t_(5)_ = 5.188, *P* < 0.05 in Ctr + veh group and t_(5)_ = 4.701, *P* < 0.05 in DIO + MK-801 group); however, rats in the DIO + veh group failed to show a preference for the target quadrant (t_(5)_ = 0.593, *P* > 0.05) (Fig. 5C). MK-801 treatments had clear effects on the number of platform crossings (F_(2,15)_ = 11.08, *P* < 0.05, Fig. 5B) and the percentage of time spent in the target quadrant (F_(2,15)_ = 5.783, *P* < 0.05, Fig. 5C). Compared with the DIO + veh group, the DIO + MK-801 group’s number of platform crossings (t_(15)_ = 3.457, *P* < 0.05) and percentage of time spent in the target quadrant (t_(15)_ = 2.926, *P* < 0.05) were markedly increased (Table 5).

### Effects of MK-801 on BDNF expression and CaMKII and CREB activation in the DG of DIO rats

**Fig. 6:**
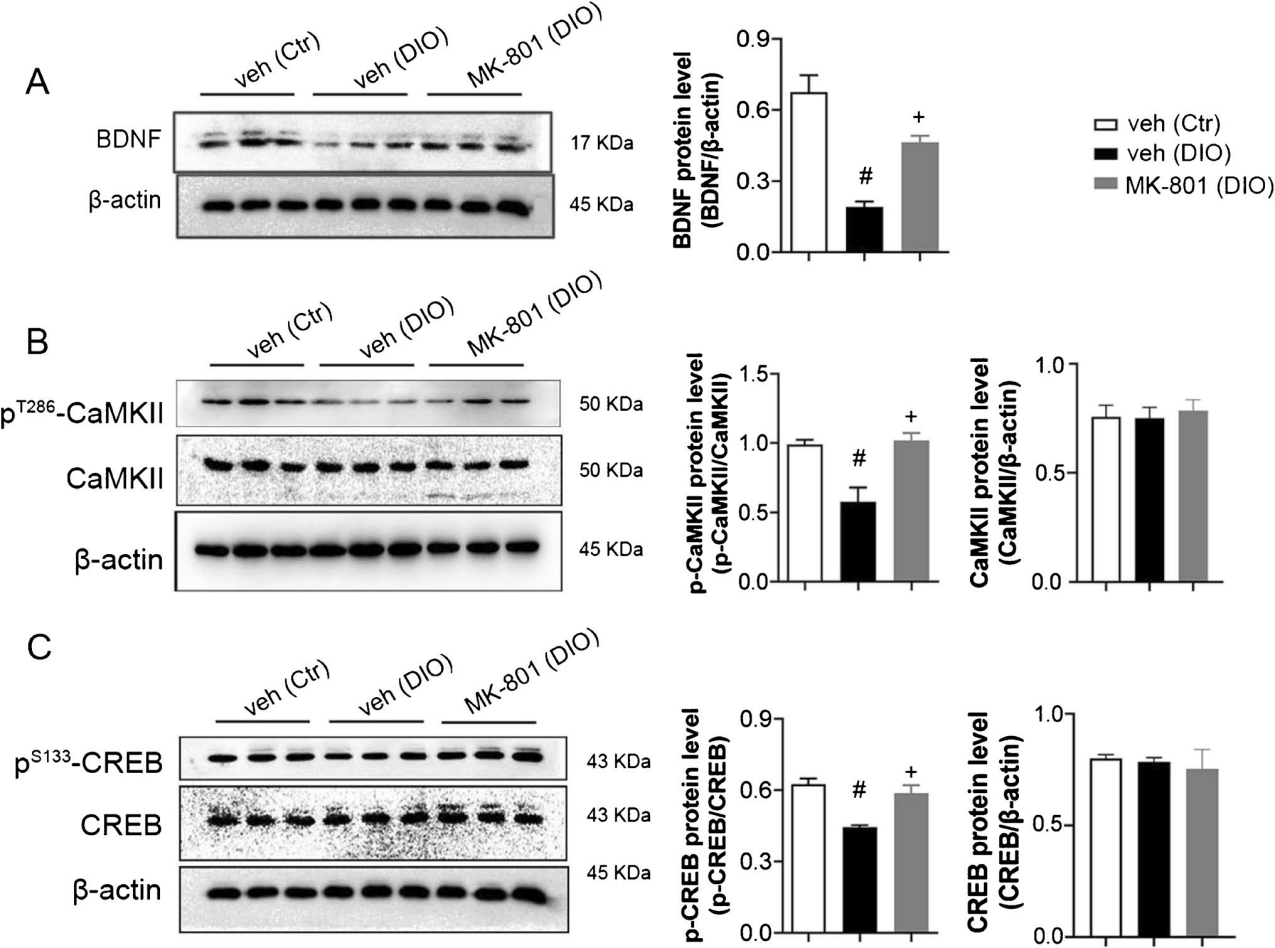
Effect of MK-801 on activation of CaMKII **A** and CREB **B** and expression of BDNF **C** in the DG in DIO rats. Data are mean ± SEM; n = 3/group. ^#^*P* < 0.05 compared with control + vehicle group, ^+^*P* < 0.05 compared with DIO + vehicle group

Finally, we observed the effects of MK-801 on BDNF expression and CaMKII and CREB activations in the DG of DIO rats (Fig. 6). MK-801 treatment had clear effects on the activation of CaMKII (F_(2,6)_ = 12.82, *P* < 0.05, Fig. 6A) and CREB (F_(2,6)_ = 16.20, *P* < 0.05, Fig. 6B) and the expression of BDNF (F_(2,6)_ 27.76, *P* < 0.05, Fig. 6C). Repeated measurements further showed that, compared with the DIO + veh group, the activation of CaMKII (t_(4)_ = 3.849, *P* < 0.05) and CREB (t_(4)_ = 4.288, *P* < 0.05) and expression of BDNF (t_(4)_ = 7.64, *P* < 0.05) of DIO + MK-801 group were markedly increased (Table 6).

**Table 6:** Effect sizes of NR2B, BDNF, p-CaMKII and p-CREB (Variable: MK-801)

Name	Cohen’s d
BDNF	6.24
p-CaMKII	3.14
p-CREB	3.50

## Discussion

We examined the involvement of Glu and NMDA receptor and the mediating molecular mechanisms in spatial learning and memory in a rat obese model. Main findings of the present study are: 1) the body weight and metabolic parameters in the serum, including CHO, leptin, and insulin, were significantly increased in diet-induced obese rats (DIO, from 4 to 12 weeks) compared to those of Ctr, confirming the establishment of DIO rat model. 2) The escape latency of DIO rats were significantly prolonged, and the percentage of time spent in the target quadrant and the number of platform crossings were reduced in the DIO rats, indicating that spatial learning and memory were impaired in this group. 3) In the DG region of the hippocampus of DIO rats, the Glu response was significantly enhanced during spatial learning, and the expression of the NMDA receptor (NR2B subunit) was significantly increased, which were prevented by MK-801, an antagonist of NMDA receptor. 4) Furthermore, BDNF expression and CaMKII/CREB activation (phosphorylation) were significantly reduced in the DG region of the hippocampus of DIO rats. 5) Microinjection of MK-801 into the DG reversed BDNF expression and CaMKII/CREB activation. Importantly, MK-801 reversed spatial learning and memory impairment in DIO rats. Taken together, these results suggest that Glu and NMDA receptor in the hippocampal DG could potentially mediate spatial learning and memory impairment in DIO rats via downregulation of the CaMKII-CREB-BDNF signaling pathway.

Physical disability and cognitive disturbance are associated with obesity in adults and adolescents, and the incidence of obesity is increasing in all age ranges, particularly in adolescents [29]. This is alarming because adolescence is a crucial period for brain development for cognition and memory [32]. The overconsumption of energy-dense, palatable foods is one of the major reasons for obesity, and evidence indicates that an energy-dense diet could be more harmful for the adolescent consumers than the adults. Overconsumption of high fat diet throughout adolescence, but not during adulthood, is associated with changes in spatial and emotional memory [5, 26]. Experimental models of DIO rats have already been used in various studies to investigate molecular mechanisms mediating cognition and memory [22, 9, 7] and our results are in line with the previous conclusions that consuming fat-rich diet is linked with spatial learning and memory deficit in rats when induced from young (4 weeks) to adult (12 weeks).

The hippocampal DG plays an important role in encoding and processing spatial information and is a critical site for adult neurogenesis, as newborn neurons in the DG contribute to spatial learning performance [25, 8]. Several lines of evidence have shown that DG lesions in rats impair the formation of spatial memory. In addition, morphological damage to the DG and a decrease in neurogenesis in this region appear in DIO rats [4, 14, 26]. Therefore, we hypothesized that the hippocampal DG is involved in the spatial cognitive deficits induced by DIO. Structurally, hippocampal DG could be linked to the learning and memory in a way that high frequency electrical stimulation or information input to penetrating fibers from the entorhinal cortex causes the presynaptic releases of Glu, which acts on AMPA receptors in the postsynaptic membrane, leading to the depolarization of postsynaptic neuron to release Mg^2+^ from the NMDA receptor. NMDA receptor activation then induces an influx of Ca^2+^ in large quantities, triggering Ca^2+^-dependent cascade reactions to form and maintain LTP [3, 19]. Previous results from our research group showed that Glu and NMDA receptors in the hippocampal DG region are involved in active and passive avoidance behavior, spatial learning, memory and relative LTP [35, 36], suggesting that Glu-NMDA receptor signaling in the DG plays an important part in the learning and the LTP. Indeed, direct injection of a non-competitive NMDA receptor antagonist, MK-801, into the hippocampus blocks Glu signaling pathway and disrupts memory consolidation of the inhibitory avoidance task in mice [12] and LTP in the DG granule cells of rats [18]. MK-801 is well known to exert allosteric regulation of target channels by acting on the benzene-ring-binding site of the receptor. In terms of NMDA receptor in the postsynaptic membrane, MK-801 can effectively block Glu binding to the NMDA receptor, reduce Ca^2+^ influx through the channel and attenuate the effects of the NMDA receptor [31]. Previously, we also showed that MK-801 administration in the DG abolished NMDA receptor activity, disrupted memory consolidation of the inhibitory avoidance task in normal rats [15]. Accordingly, we speculate that MK-801 injection in DG blocks NMDA receptor and affects spatial learning and memory in normal rats. Under physiological conditions, precise regulation of Glu-NMDA receptor signaling in DG is essential molecular mechanisms of learning and memory. An increase or decrease of Glu-NMDA receptor response may negatively affect learning and memory performance [24, 13]. For example, Glu increment in DG was shown to be associated with the impairment of learning and memory in Alzheimer rat model [28]. In contrast, vascular dementia is linked with Glu reduction in DG [20]. The results of this study also showed that, in the DG region of the hippocampus of DIO rats, the Glu response was significantly enhanced during spatial learning, and the expression of the NMDA receptor (NR2B subunit) was significantly increased. Experimental results with local injection of MK-801 to block NMDA receptor in the DG area of the hippocampus of DIO rats significantly ameliorated the spatial learning and memory impairments. Collectively, upregulation of Glu-NMDA receptor transmission in the DG is associated with spatial learning and memory impairment in DIO rats.

CaMKII is highly abundant brain protein which is activation by Ca^2+^ following NMDA receptor upregulation in the postsynaptic density [30]. Among multiple NMDA receptor subunits, the affinity between NR2B and CaMKII is significantly higher than that of other subunits. The NR2B-CaMKII complex in the dense region [2] are involved in the promotion of the expression of learning- and memory-related molecules, such as BDNF, by activating the transcription factor CREB. As such, CaMKII-CREB-BDNF axis is important in promoting the formation and maintenance of hippocampal learning and memory [33]. The stoichiometry of NR2B and CaMKII the complex homeostasis in the DG of hippocampus are considered important molecular basis for the learning and memory. Alterations of the expressions and the disruption of NR2B/CaMKII homeostasis in the DG are associated with abnormal functions. Under pathological conditions, although NR2B expression significantly increases, the proportion of NR2A/B phosphorylation in the body significantly decreases, leading to the inhibition of CaMKII activity and subsequent impairment of learning, memory, and related LTP formation [6]. The results of this experiment also found that the BDNF expression and phosphorylated fraction and activation of CaMKII and CREB were significantly reduced in the DG region of the hippocampus of DIO rats. MK-801 antagonization of the NMDA receptor significantly increased CaMKII and CREB activation and BDNF expression in the DG, supporting the mechanistic involvement of NDMA receptor, CaMKII and CREB activation with BDNF expression in spatial learning and memory impairments of DIO rats.

In conclusion, we provide direct evidence to suggest that Glu and NMDA receptor in the hippocampal DG are upregulated in DIO rats which could be linked to spatial learning and memory impairment in DIO rats. Downregulation of the CaMKII, CREB and BDNF signaling pathways may mediate the hippocampal DG dysfunction.

## Data Availability

Datasets used in the study can be accessed from the first, second and corresponding authors.
